# Ehlers–Danlos syndrome, an underreported consideration in plastic surgery: A narrative review of cases

**DOI:** 10.1016/j.jpra.2026.07.027

**Published:** 2026-07-24

**Authors:** Colton D. Brown, Stanley J. Hickman, Tatjana Mortell, Simran K. Chandawarkar, Subin Cho, Rahim Nazerali

**Affiliations:** a University of Florida College of Medicine, Gainesville, FL, USA; b Mercer University School of Medicine, Macon, GA, USA; c Department of Surgical Education, Orlando Health, Orlando, FL, USA; d Northeast Ohio Medical University, Rootstown, OH, USA; e Loyola University Chicago Stritch School of Medicine, Maywood, IL, USA; f Department of Plastic Surgery, Stanford University School of Medicine, Stanford, CA, USA

**Keywords:** Ehlers–danlos syndrome, Plastic surgery, Wound healing, Tissue fragility, Surgical outcomes, Narrative review

## Abstract

**Background:**

Ehlers–Danlos Syndrome (EDS) encompasses a variety of collagen disorders characterized by joint hypermobility, skin laxity, and tissue fragility. Evidence to guide plastic surgeons for both elective and reconstructive procedures is limited and consists predominantly of case reports and series. As higher–level comparative data are scarce, synthesizing case–level data remains essential to understand surgical risks, considerations, and postoperative outcomes in this patient population.

**Methods:**

A narrative review of case reports and series was conducted using PRISMA guidelines across Scopus, PubMed/MEDLINE, Cochrane, Web of Science, and Embase. Studies from inception to September 2025 were screened for surgical management of patients with EDS. After screening 1816 abstracts with review of 230 full texts, 52 studies met inclusion criteria. Data were collected on demographics, EDS subtype, surgical indication, procedures, perioperative care, and complications. Case–level data were summarized descriptively as counts and percentages, with continuous variables reported as mean (SD) and median (range).

**Results:**

The 52 studies described 77 individual cases of EDS, most frequently hypermobile (36.4%), classical (27.3%), and vascular (9.1%) subtypes. The cohort was predominantly female (female–to–male ratio 1.8:1), with a mean age of 31.9 ± 16.6 years (median 28, range 3–64). Reconstruction was the primary indication (67.5%), with additional representation from aesthetic (13.0%). Across case reports and series, frequently discussed strategies included tension–free layered closure, vascular preservation, gentle tissue handling, adhesive supports, and prolonged suture retention.

**Conclusions:**

Themes emerged regarding optimal practices for patients with EDS. The techniques emphasized attention to tension reduction, vascular friability, and postoperative supports to mitigate complications in this patient demographic. Heterogeneous reporting in the literature limits the generalizability of findings and underscores the need for standardized documentation. However, these cases represent a potential for developing perioperative strategies and wound–care protocols to guide best practices in this complex population.

## Introduction

Ehlers–Danlos Syndrome (EDS) is a group of connective tissue genetic diseases characterized by joint hypermobility, skin hyperextensibility, and fragility of tissue. Thirteen clinical and genetic subtypes have been described, with several of widespread clinical relevance. The most common subtypes of EDS encountered in the operating room are classical, hypermobile, and vascular EDS, each with varying degrees of perioperative risk. Hypermobile EDS is associated primarily with joint hypermobility and relatively lower surgical risks, while classical EDS is characterized by increased soft tissue fragility and impaired wound healing.^1^, ^2^, ^3^, ^4^, ^5^ The vascular subtype carries a substantially higher operative risk, particularly a potential for life–threatening vascular and organ ruptures, therefore warranting heightened perioperative caution.^1^, ^2^, ^3^, ^4^, ^5^ These patients often consult plastic and reconstructive surgeons for the management of joint and skin laxity, atrophic scarring, and other connective tissue abnormalities. Plastic and reconstructive surgeons are more often consulted for other primary surgical reasons rather than elective corrections of tissue abnormalities in this patient population. This arises mainly as perioperative management and complication mitigation. Common consults include wound closure and soft–tissue repair when tissue fragility is a concern.

Underdocumentation in the surgical literature is a well–recognized issue, reflected in inadequate outcome reporting, limited protocol availability, and inconsistent definitions of complications.^6^, ^7^ There exists a limit of comparative studies with a predominance of case reports and series which still offer experience to learn from. While this problem is not unique to plastic and reconstructive surgery, its widespread presence across surgical disciplines, especially when considering EDS–specific surgical challenges, has serious clinical implications. These include increased perioperative risk, insufficient preoperative planning, and missed opportunities for multidisciplinary care. Such underdocumentation might lead to inappropriately performed surgery, inadequate hemostatic control, and perpetuation of misconceptions that EDS is an absolute contraindication to elective surgery. Additionally, underrecognition of EDS has implications of delayed diagnosis, repeated surgical failure, and hindrance in the development of evidence–based, standardized guidelines for this complex patient population.^8^, ^9^, ^10^

Historically, EDS has been considered a relative contraindication for elective plastic surgery due to concerns of wound dehiscence, bleeding, and impaired healing. However, emerging evidence suggests that elective procedures, such as scar revision, herniotomy, and soft tissue reconstruction, may be safely performed when appropriate precautions are taken. Particularly in patients with hypermobile EDS, complication rates were noted to be comparable to those of non–EDS controls.^1^^,^^5^^,^^11^

Operative risk in EDS is primarily driven by tissue and vascular fragility, impaired wound healing, and increased bleeding tendency, with heightened concern in classical and vascular subtypes.^1^^,^^5^^,^^11^ Successful outcomes are associated with specific intraoperative strategies, including gentle tissue handling, meticulous hemostasis, and tension–free closure using non–absorbable or slowly absorbable monofilament sutures in a layered fashion. Additional considerations include mesh reinforcement when undergoing abdominal wall closure, hematology consultation in vascular EDS, and vigilant postoperative wound monitoring.^1^^,^^5^^,^^12^, ^13^

Given the unique inherent risks associated with EDS, it is imperative to develop a framework for evaluating surgical candidacy to optimize outcomes for individual patient disease subtype and risk profile. The objective of this review is to synthesize published cases that detail perioperative strategies and outcomes in plastic and reconstructive procedures among patients with EDS, a topic that remains underreported in a comprehensive manner. We specifically aim to characterize the types of operative procedures, closure and suture techniques, wound care management, and outcomes. Through this synthesis, we aim to provide a practical reference for surgeons and contribute to the development of evidence–based recommendations for surgical management of this complex patient population.

## Methods

A narrative review was conducted in accordance with PRISMA guidelines using the electronic databases Scopus, PubMed/MEDLINE, Cochrane, Web of Science, and Embase. The search was limited to English–language, peer–reviewed publications from inception to September 2025. Search terms were developed in collaboration with health science librarians at Tulane University School of Medicine. These included: “Ehlers–Danlos syndrome AND plastic surgery,” “Ehlers–Danlos syndrome AND reconstructive surgery,” “Ehlers–Danlos syndrome AND aesthetic surgery,” “Ehlers–Danlos syndrome AND cosmetic surgery,” “Ehlers–Danlos syndrome AND surgery,” “Ehlers–Danlos syndrome AND graft,” “Ehlers–Danlos AND flap,” “Ehlers–Danlos AND free tissue,” “Ehlers–Danlos AND laser,” and (“Ehlers–Danlos syndrome” OR “EDS”) AND (“plastic surgery” OR “reconstructive surgery” OR “cosmetic surgery”) AND (“suture techniques” OR “wound care” OR “closure techniques”) AND (“surgical outcomes” OR “complications” OR “wound healing”).

Titles and abstracts were screened independently by a minimum of two reviewers. Conflicting responses or disagreements regarding study inclusion were resolved by a third reviewer. Full–text review and data extraction were conducted for studies meeting predefined inclusion criteria. Studies were preliminarily excluded if they were outside the scope of plastic surgery, referenced conditions other than EDS, or were not available in English translation. Remaining studies were included if they addressed the surgical or plastic surgical management of patients with any subtype of EDS, evaluated outcomes in plastic surgery patients with EDS, or discussed complications or technical considerations specific to this population. Eligible study designs included clinical trials, cohort studies, case–control studies, retrospective reviews, case reports and series, and prior systematic reviews. Only studies discussing plastic surgical interventions were retained.

Following de–duplication and screening, a total of 52 unique records were identified which met the final inclusion criteria. The unit of analysis was the individual EDS case within the plastic and reconstructive surgical scope, defined as a single operative episode and including both operatively and non–operatively managed patients. Studies reporting individual case reports, case series, and single–arm cohorts were pooled at the patient level (n = 77), whereas four comparative cohort studies reported only aggregate outcomes and were summarized separately and excluded from the pooled cohort (Fodor 2025, Najafian 2022, Norman 2022, and Lu 2020). Per–case data for all included studies are provided in Supplementary Table S1. Data extracted included patient demographics (age, sex, EDS subtype), anatomical region, indication (aesthetic, reconstructive, or other), procedure performed, preoperative optimization, intraoperative techniques (e.g., tissue handling, closure methods), postoperative care (e.g., dressings, wound care), and complications. Data were extracted using Covidence and stored in a shared Google Sheets document.

Given the heterogeneity of included studies, outcomes were summarized descriptively. Reported complications, surgical outcomes, and perioperative management strategies were extracted. Categorical variables are presented as counts and percentages, and continuous variables as mean (SD) and median (range); no inferential analyses were performed.

## Results

### Patient demographics

**Fig. 1:**
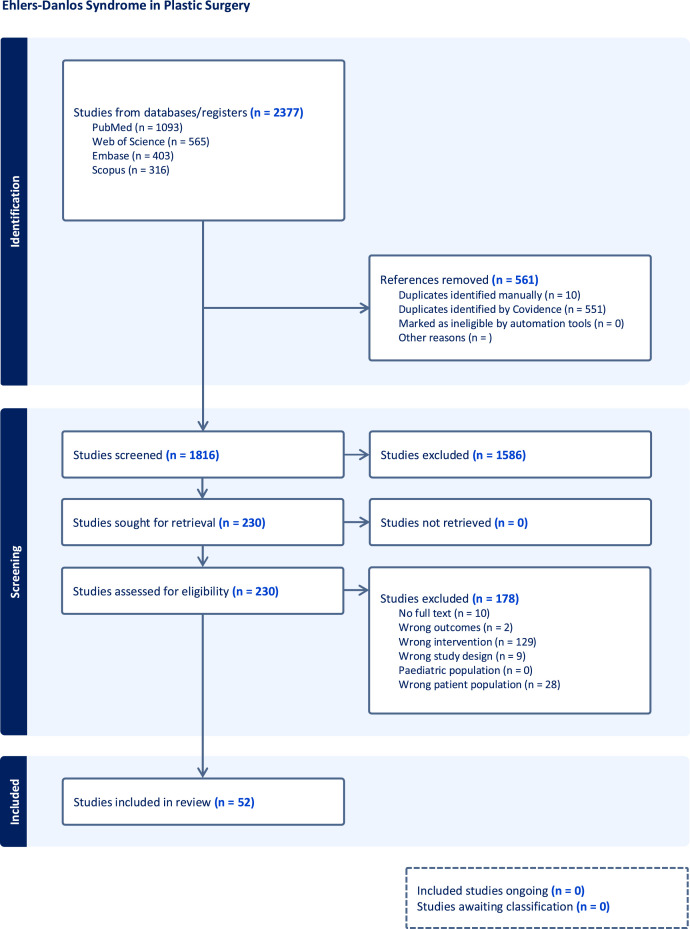
PRISMA flow diagram illustrating the study selection process for inclusion in the narrative review.

A total of 52 studies met inclusion criteria (Table 1), comprising 77 individual patients with EDS across plastic surgery subspecialties, as higher–level evidence remains scarce.^14^, ^15^, ^16^, ^17^, ^18^, ^19^, ^20^, ^21^, ^22^, ^23^, ^24^, ^25^, ^26^, ^27^, ^28^, ^29^, ^30^, ^31^, ^32^, ^33^, ^34^, ^35^, ^36^, ^37^, ^38^, ^39^, ^40^, ^41^, ^42^, ^43^, ^44^, ^45^, ^46^, ^47^, ^48^, ^49^, ^50^, ^51^, ^52^, ^53^, ^54^, ^55^, ^56^, ^57^, ^58^, ^59^, ^60^, ^61^, ^62^, ^63^, ^64^, ^65^, ^66^ The study selection process is summarized in the PRISMA flow diagram (Fig. 1). Most patients underwent an operative or procedural intervention in 65 (84.4%), whereas 12 (15.6%) were managed non–operatively. Female patients were more frequently represented than male patients (female 45 [64.3%], male 25 [35.7%]; F:M 1.8:1) among the 70 cases with sex reported; sex was not reported in 7 cases. Patient age ranged widely, averaging 31.9 ± 16.6 years (median 28, range 3–64); 14 cases were pediatric (<18 years), and age was not reported in 14 cases. The most frequently documented EDS subtypes were hypermobile (28 [36.4%]), classical (21 [27.3%]), and vascular (7 [9.1%]), followed by dermatosparaxis (6 [7.8%]), arthrochalasia (2 [2.6%]), kyphoscoliotic (1 [1.3%]), and combined subtypes (2 [2.6%]); the subtype was not specified in 10 cases (13.0%) (Table 2).

**Table 1:** Summary of published studies reporting plastic and reconstructive surgical management of patients with Ehlers–Danlos syndrome. Per–patient detail (age, sex, procedure, perioperative considerations, and complications) is provided in Supplementary Table S1.^14^, ^15^, ^16^, ^17^, ^18^, ^19^, ^20^, ^21^, ^22^, ^23^, ^24^, ^25^, ^26^, ^27^, ^28^, ^29^, ^30^, ^31^, ^32^, ^33^, ^34^, ^35^, ^36^, ^37^, ^38^, ^39^, ^40^, ^41^, ^42^, ^43^, ^44^, ^45^, ^46^, ^47^, ^48^, ^49^, ^50^, ^51^, ^52^, ^53^, ^54^, ^55^, ^56^, ^57^, ^58^, ^59^, ^60^, ^61^, ^62^, ^63^, ^64^, ^65^, ^66^

Author / Year	No. of cases	Anatomical region	Indication	Key conclusions / Takeaways
Ricketson (1957)	1	Lower Extremity	Reconstructive	Split–thickness skin grafting can achieve full take with normal donor–site healing in EDS, but scar excision requires tension–free multilayer closure.
Reidy (1963)	7	Multisite	Aesthetic; Reconstructive	In classical EDS, scars can be improved by excision and layered closure, but deep dermal sutures hold poorly; mattress sutures without undermining, prolonged suture retention of about three weeks, and adhesive support are therefore recommended.
Beighton & Bull (1970)	1	Lower Extremity	Aesthetic	Operative risk is subtype–dependent, and surgery is contraindicated in the vascular (ecchymotic) type; tissue fragility is countered with electrocautery, retention sutures tied at a distance from the incision, adhesive–strip support, and immobilization of the part, with scar excision timed to early adolescence.
Guerrerosantos & Dicksheet (1985)	1	Craniofacial	Aesthetic	Rhytidoplasty is best discouraged in EDS; exclude the diagnosis preoperatively in facelift candidates, and if surgery proceeds, undermine and excise skin only without plication and type and crossmatch blood preoperatively.
Moore et al. (1985)	2	Hand/Upper Extremity	Reconstructive	In EDS, predictable bony procedures may be preferable to soft–tissue reconstruction for small–joint instability; conservative management is appropriate for less severe carpometacarpal instability.
Borris et al. (1997)	1	Craniofacial	Reconstructive	With meticulous technique, sutures placed away from the wound edge, and patience, split–thickness skin grafting can succeed in EDS, though donor–site healing rather than graft take is the limiting factor.
Erçöçen et al. (1997)	1	Hand/Upper Extremity	Reconstructive	Dynamic swan–neck deformity from ligamentous laxity was splinted rather than reconstructed, since the subtype's tissue fragility and laxity make surgery hazardous and unreliable
Oliver et al. (2000)	6	Multisite	Other	Local anesthetic resistance in EDS is not due to rapid tissue dispersal; increasing dose is unlikely to overcome resistance
Badia et al. (2005)	1	Hand/Upper Extremity	Reconstructive	Arthroscopic tendon interposition arthroplasty with thermal capsular shrinkage can restore stability and function in EDS–related thumb CMC arthritis.
Mueller et al. (2005)	1	Craniofacial	Aesthetic	In classical EDS, less–invasive full–face laser resurfacing achieved better wrinkle reduction than prior conventional facelifts, with the CO_2_/erbium:YAG combination laser healing faster than the CO_2_ laser alone.
Rossi & Kappel (2006)	1	Craniofacial	Reconstructive	TMJ reconstruction with a vascularized temporalis osteofascial flap fixed bone–to–bone avoids reliance on the poor soft–tissue healing seen in EDS.
Tan et al. (2009)	1	Craniofacial	Reconstructive	EDS–related impaired wound healing and anesthetic risk can make cleft repair hazardous, and conservative management may be appropriate through shared decision–making.
Whitaker et al. (2009)	1	Trunk/Abdomen	Reconstructive	Recommend plastic surgery in preoperative planning for any EDS type VII surgery; meticulous low–tension closure with tension–offloading support, prolonged dermal taping, and strict bleeding precautions achieved uncomplicated healing despite severe skin fragility.
Cheung et al. (2010)	1	Hand/Upper Extremity	Reconstructive	In classic EDS, trigger fingers were caused by intratendinous fraying of the tendons catching the A1 pulley; pulley–sparing debridement of the frayed fibers, with avoidance of the precipitating overuse produced a good outcome and no recurrence at two years, suggesting A1 pulley release may be unnecessary in EDS.
Kumar et al. (2010)	1	Lower Extremity	Reconstructive	Intermittent negative–pressure dressing overcame prior delayed healing of a grafted leg wound, with the graft showing reduced primary contraction.
de Weerd et al. (2012)	1	Trunk/Abdomen	Reconstructive	A complex abdominal wall hernia in vascular EDS was successfully managed with staged, tension–free reconstruction using a retromuscular double polypropylene mesh and temporary negative–pressure stabilization of the abdominal wall, with no recurrence at two years.
de Weerd et al. (2012)	1	Trunk/Abdomen	Reconstructive	Deepithelialized muscle–sparing TRAM flap can serve as a vascularized "dam" filling the pelvic inlet to treat enterocele, avoiding synthetic mesh and exploiting well–vascularized autologous tissue better suited to impaired EDS healing, with little donor–site morbidity.
Kelley & Heller (2012)	1	Trunk/Abdomen	Reconstructive	In EDS patients bridge fascial defects with biologic mesh and close skin with barbed sutures placed perpendicular to the wound to diffuse tension over a wide surface rather than at the edges, preventing tearing and recurrent dehiscence
Ryan et al. (2012)	1	Trunk/Abdomen	Reconstructive	Early recognition of EDS in unexplained wound breakdown prevents repeated invasive procedures and enables successful conservative wound management.
Bouhassira et al. (2014)	1	Breast; Trunk/Abdomen	Aesthetic	Body–contouring surgery in EDS carries high complication risk, chiefly wound dehiscence and hematoma, with only modest aesthetic gains, but may be justified in select patients when paired with tension–free multilayer closure, meticulous hemostasis, and thorough counseling.
Busch et al. (2014)	1	Lower Extremity	Reconstructive	In kyphoscoliotic EDS, a non–healing groin wound after vascular surgery was salvaged with negative–pressure therapy and meshed split–thickness mesh skin grafting.
Krijgh et al. (2014)	6	Hand/Upper Extremity	Reconstructive	In hypermobile EDS, hemi–extensor carpi radialis brevis tenodesis stabilized the midcarpal joint and reduced pain and orthotic dependence across five patients, with one major recurrence requiring revision; autologous tendon reconstruction is feasible despite abnormal collagen.
Creasy et al. (2016)	1	Breast	Reconstructive	Classical EDS is not a contraindication to microsurgical free–flap (DIEP) breast reconstruction; delicate handling with minimal needle passes gave uneventful anastomoses and healing without dehiscence or atrophic scarring; donor–site hernia suggests patients may benefit from routine mesh reinforcement.
Harrison et al. (2016)	—	Trunk/Abdomen	Reconstructive	EDS predisposes to abdominal wall hernia and to high recurrence through defective collagen processing, though high–level evidence is lacking; the authors advise optimizing repair with mesh and local tissue.
Lindsay et al. (2016)	1	Craniofacial	Reconstructive	EDS carries a high bleeding risk despite normal standard coagulation tests; a preoperative bleeding workup with platelet aggregometry is recommended, with prophylaxis consisting of antifibrinolytics, DDAVP (± platelet transfusion), and recombinant factor VIIa reserved for severe bleeding, alongside atraumatic technique and avoidance of NSAIDs, anticoagulants, and antiplatelets.
Rollett et al. (2016)	1	Craniofacial	Aesthetic	Facelifts can be performed safely in classical EDS using a modified MACS technique with no skin undermining that reduces invasiveness and transfers tension to deep sutures rather than skin, preventing scar widening; generalized vessel fragility nonetheless persists.
Ericson & Wolman (2017)	—	Multisite	Reconstructive	Orthopedic surgery in EDS should be selective and diagnosis–driven; non–operative care is preferred, but targeted stabilization or nerve decompression may be effective when conservative measures fail.
Joseph et al. (2017)	—	Craniofacial	Aesthetic; Reconstructive	Classic and hypermobile EDS can usually undergo elective facial plastic surgery, but vascular EDS is contraindicated. Screen and refer for genetic subtyping, and obtain cardiovascular clearance, crossmatch, and coagulopathy screening preoperatively. Intraoperatively, handle tissue atraumatically with minimal retraction, secure hemostasis with electrocautery while avoiding vessel loops and clamps, close in layers with generous deep tension–minimizing sutures and adhesive tape, avoid skin staples, and leave nonabsorbable sutures in place twice as long.
Hosomi et al. (2018)	1	Lower Extremity	Reconstructive	Net–pattern small–slice split–thickness skin graft harvesting enables safe donor–site healing and repeat grafting in fragile EDS skin. Manual freehand dermatome harvesting lets the surgeon adjust pressure to avoid deeper–than–planned cuts.
Baik et al. (2019)	1	Lower Extremity	Reconstructive	In EDS, bolster sutures should be avoided because they impair perfusion; tension–free closure with mesh overlay support and delayed suture removal are preferred, and a collagen–alginate dressing may be considered to accelerate healing.
Hevesi et al. (2019)	9	Hand/Upper Extremity	Reconstructive	Conservative management kept pain stable but range of motion declined over time, while surgery gave significant durable pain relief (severe pain 83% to 0%) and trended toward stronger grip; most operative cases added concurrent ligament reconstruction to address the underlying laxity.
Schneider et al. (2019)	1	Hand/Upper Extremity	Reconstructive	Use of tendon allograft ligament reconstruction over the patient's pathologic native tissue, with a temporary hinged internal fixator, can restore stability and preserve function in EDS–related elbow instability.
Shaharan et al. (2019)	1	Lower Extremity	Reconstructive	In fragile EDS skin, combining adhesive strips with sutures reduces shear forces and prevents cheese–wiring.
Xu et al. (2019)	1	Craniofacial	Aesthetic	Careful facial scar revision with tension–minimizing, precisely apposed layered closure can yield favorable scar outcomes in classical EDS.
Lu et al. (2020)	19^b^	Craniofacial	Reconstructive	Myofascial flap closure cut overall reoperation after craniocervical fusion without added wound complications; in EDS patients the reduction was only a nonsignificant trend, and they remain at higher reoperation risk than non–EDS patients.
Scheufler et al. (2020)	2	Trunk/Abdomen	Reconstructive	In classic EDS, mesh–reinforced abdominal wall reconstruction with rectus plication relieved disabling functional symptoms without major complications.
Deshpande et al. (2021)	1	Breast	Reconstructive	Reinforcing a de–epithelialized Goldilocks mastectomy with acellular dermal matrix is proposed as a barrier to skin breakdown that supports a later implant, suiting EDS–related poor wound healing.
Najafian et al. (2022)	36^a^	Multisite	Reconstructive	EDS patients had major–complication and reoperation rates comparable to controls and significantly fewer wound–healing issues, supporting that non–vascular EDS should not be a barrier to reconstructive surgery.
Norman et al. (2022)	34^b^	Craniofacial	Reconstructive	In EDS patients undergoing craniocervical junction surgery, vascularized myofascial flap closure reduced complications after complex surgery to the level of simple surgery and yielded a zero percent cerebrospinal fluid fistula rate, supporting it as a safe option in this high–risk population.
Padmanabhan et al. (2022)	1	Breast	Reconstructive	Local mechanical stress from the textured implant overrides the genetic collagen defect and drives prolonged peri‑implant inflammation; managed with complete capsulectomy and smooth–implant exchange.
Hasegawa et al. (2023)	1	Lower Extremity	Reconstructive	Pedicled posterior tibial artery perforator flap reconstruction can be performed successfully in vascular EDS with preoperative perforator mapping and perforator–sparing dissection.
Ishimoto et al. (2023)	1	Breast	Reconstructive	Bilateral Wise–pattern, inferior–pedicle reduction mammaplasty in an adolescent with hypermobile EDS healed without complications and relieved macromastia symptoms, suggesting hypermobile EDS is not an absolute contraindication to the procedure
Lellouch et al. (2023)	1	Trunk/Abdomen	Reconstructive	Two–stage free flap reconstruction can safely restore full–thickness abdominal wall defects in vascular EDS while minimizing catastrophic risk
Shomorony et al. (2023)	1	Craniofacial	Reconstructive	Septorhinoplasty can be performed successfully in EDS with an endonasal approach and durable closure (polyglactin quilting sutures, septal splints, prolonged suture retention); multidisciplinary perioperative management is recommended.
Wanderley et al. (2023)	1	Craniofacial	Reconstructive	The authors emphasize meticulous, atraumatic soft–tissue handling with taper–point needles, a multidisciplinary approach with plastic surgery, and a low threshold for early magnetic resonance imaging given the diagnostic delay caused by an atypical presentation.
Angwin et al. (2024)	—	Multisite	Other	Injury prevention plus tension–free layered closure, prolonged suture retention, least–reactive monofilament, cyanoacrylate for small wounds, low–adherence dressings, and bleeding precautions are critical due to the intrinsic skin fragility and impaired healing in EDS.
Morris et al. (2024)	4	Breast	Aesthetic; Reconstructive	Hypermobile EDS is not a contraindication to breast reduction or mastopexy, which yielded no wound–healing or scarring complications with uniform pain relief and satisfaction.
Nehila et al. (2024)	1	Breast	Reconstructive	Two–stage tissue–expander reconstruction can be safely performed in combined classical EDS and vascular EDS with careful planning.
Angwin et al. (2025)	5	Multisite	Reconstructive	In dermatosparaxis EDS, sutures should be minimized in favor of adhesive strips and cyanoacrylate glue, with double–layered tension–free closure when needed; redundant skin folds often require uncomplicated debulking; and early plastic surgery referral with careful manual handling is recommended.
Fodor et al. (2025)	100^a^	Multisite	Aesthetic; Reconstructive	EDS patients had complication rates no higher than controls supporting that EDS, particularly hypermobile EDS, should not be treated as a blanket contraindication to elective plastic surgery.
Rostami et al. (2025)	1	Lower Extremity	Reconstructive	In classical EDS, a large complex knee wound was closed with a meshed split–thickness skin graft plus Recell autologous skin–cell spray, achieving 99% take with minimal donor morbidity.
Ziółkowska et al. (2025)	1	Multisite	Reconstructive	Extensive burns in EDS demand prolonged, multi–stage reconstruction combining acellular dermal matrix, MEEK–expanded split–thickness grafts, and cultured autologous skin cells, with laser speckle contrast analysis for perfusion monitoring.

a Retrospective cohort/comparative studies (Najafian 2022, n = 36; Fodor 2025, n = 100) reporting EDS outcomes against non–EDS controls; summarised, not included in the individual–case count.

b Lu (2020; 19 EDS patients of 24) and Norman (2022; 34 EDS patients of 62) report overlapping cohorts from one institution; summarised as study–level entries and not included in the individual–case count.

**Table 2:** Demographic and clinical characteristics of pooled cohort (n = 77).

Characteristic	n (%)
**Sex (n=70 reported)**	
Female	45 (64.3%)
Male	25 (35.7%)
Not reported	7
**Age, years (n=63 reported)**	
Mean (SD)	31.9 (16.6)
Median (range)	28 (3–64)
**EDS subtype (N=77)**	
Hypermobile (hEDS)	28 (36.4%)
Classical (cEDS)	21 (27.3%)
Vascular (vEDS)	7 (9.1%)
Dermatosparaxis (dEDS)	6 (7.8%)
Arthrochalasia (aEDS)	2 (2.6%)
Kyphoscoliotic (kEDS)	1 (1.3%)
Combined	2 (2.6%)
Not specified	10 (13.0%)
Anatomical region (N=77 cases)*	
Hand/upper extremity	24 (31.2%)
Craniofacial	14 (18.2%)
Lower extremity	11 (14.3%)
Breast	10 (13.0%)
Multisite	10 (13.0%)
Trunk/abdomen	9 (11.7%)
Not specified	1 (1.3%)
**Surgical indication (N=77)**	
Reconstructive	52 (67.5%)
Aesthetic	10 (13.0%)
Other	6 (7.8%)
No surgical indication	9 (11.7%)
**Operative status (N=77)**	
Operative	65 (84.4%)
Non–operative	12 (15.6%)
**Complications (≥1 reported)**	
All cases	32/77 (41.6%)
Operative cases only	31/65 (47.7%)

Data are n (%); sex and age percentages use cases with available data (n = 70 and n = 63), all others N = 77.

*Two cases spanned two regions (regional total 79). "Combined" = two concurrent subtypes.

### Anatomical regions and procedures

Cases spanned multiple anatomical regions. The most frequently involved were the hand/upper extremity (24 [31.2%]), craniofacial region (14 [18.2%]), lower extremity (11 [14.3%]), breast (10 [13.0%]), multisite (10 [13.0%]), and trunk/abdomen (9 [11.7%]); the region was not specified in 1 case (1.3%). Two cases involved two regions each, so regional counts total 79. The distribution of anatomical regions is illustrated in Table 2. Reconstructive procedures represented the majority of cases, and the operations most commonly reported were scar revision, skin grafting, rhytidectomy, breast reconstruction, and joint stabilization (e.g., MCP fusion, ligament reconstruction). By indication, most cases were reconstructive (52 [67.5%]), followed by aesthetic (10 [13.0%]) and other (6 [7.8%]); the remaining 9 (11.7%) had no surgical indication (untreated relatives or conservatively managed patients). The six 'other' cases derived from a single study of local–anesthetic efficacy (Oliver 2000).

### Preoperative optimizations

A limited portion of the cases reviewed described any form of preoperative optimization. These included specialist referrals (e.g., cardiology, rheumatology, hematology), physical rehabilitation or therapy, and risk counseling preoperatively.^14^^,^^15^

Multidisciplinary preoperative evaluation was employed in several cases involving patients with EDS. For example, patients undergoing breast reconstruction were evaluated by rheumatology and cardiology services prior to reconstruction due to established vascular compromise and thoracic root dilation.^16^ Hematology consultation was also utilized to establish a perioperative hemostatic plan with tranexamic acid and recombinant factor VIIa in rhinoplasty.^17^ Physical medicine and rehabilitation (PM&R) and physical therapy were utilized in some cases, particularly in breast surgical and oncology environments, to assess mobility restriction and assist with postoperative rehabilitation planning.^15^^,^^18^ In one case, preoperative counseling was emphasized to ensure the patient understood the elevated risks associated with surgery, including poor wound healing and tissue fragility.^14^

### Tissue handling

Among the 65 operative cases, the most frequently reported EDS–specific optimizations were tension–free or multilayer closure (13 [20.0%]), immobilization or support garments (13 [20.0%]), atraumatic tissue handling (11 [16.9%]), meticulous hemostasis (11 [16.9%]), and staged procedures (11 [16.9%]), followed by specific suture or needle choice (9 [13.8%]), skin–graft technique optimization (8 [12.3%]), and adhesive–strip support (8 [12.3%]) (Table 3). Intraoperative tissue handling was described in several studies, reflecting the well–documented concern for connective tissue fragility in patients with EDS. Shared strategies included minimizing tension and undermining, use of vascularized flaps, avoiding multiple needle passes, and gentle dissection and hemostasis.^19^^,^^20^ In a case of temporomandibular joint reconstruction, a temporalis osteofascial flap was used to provide a bone substrate for suturing attachment to avoid direct tension on soft tissues.^19^ Similarly, in a case of craniocervical instability, myofascial flap closure was utilized to reduce reoperation by providing robust, well–vascularized coverage of hardware.^21^ This technique was echoed in a posterior fossa decompression and occipitocervical fusion, where trapezius flaps were advanced to protect the occipital plate and hardware.^22^

**Table 3:** Reported EDS–specific treatment optimizations among operative cases.

Optimization category	n (% of operative)
Tension–free / multilayer closure	13 (20.0%)
Immobilization / splinting / support garment	13 (20.0%)
Atraumatic / gentle tissue handling	11 (16.9%)
Meticulous hemostasis / bleeding prophylaxis	11 (16.9%)
Staged / multistage procedure	11 (16.9%)
Skin–graft technique optimization	8 (12.3%)
Adhesive–strip / tape support	8 (12.3%)
Specific suture / needle choice	9 (13.8%)
Specialized / prolonged dressing	6 (9.2%)
Vascularized / autologous tissue (flap)	6 (9.2%)
Prolonged suture retention / delayed removal	5 (7.7%)
Mesh / ADM reinforcement	4 (6.2%)
Negative–pressure wound therapy	4 (6.2%)
Multidisciplinary / preoperative workup	4 (6.2%)
Sutures placed away from wound edge	3 (4.6%)
Avoid / limit undermining	2 (3.1%)
Avoid skin staples	0 (0.0%)
Other (procedure–specific / adjunctive)	34 (52.3%)
None / not specified	13 (20.0%)

Data are operative cases reporting each optimization (n = 65); categories are not mutually exclusive, so percentages sum to >100%. "None/not specified" = no EDS–specific optimization reported; "Other" = procedure–specific or adjunctive measures. ADM, acellular dermal matrix.

In one facelift procedure, the surgeon avoided undermining the skin flap entirely, opting instead for a modified MACS lift in an effort to decrease tension and preserve vascularity.^14^ A case of septorhinoplasty employed 4–0 polyglactin 910 quilting sutures instead of chromic gut, due to the patient’s known history of delayed healing and the need to minimize potential trauma.^23^ During a case of rhytidoplasty, undermining was avoided altogether, as the tissue could not tolerate traction forces and significant bleeding was encountered due to vascular fragility.^24^ Vascular fragility was especially concerning in microsurgical contexts. One report described limiting multiple needle passes during anastomosis to prevent intimal damage and emphasized the importance of tension–free multilayered closure.^20^ In a breast reconstruction case, intraoperative positioning and pressure management were critical to avoid pressure–related trauma.^18^ In a case of left mastectomy and axillary lymph node dissection, the Goldilocks technique was used in an effort to preserve dermal vascularity and minimize the tension during inset.^15^ In another case, deep catgut sutures could not be placed due to extreme dermal fragility; instead, subcuticular and continuous mattress were used to achieve secure closure.^25^

A particularly novel technique was described in a case of wound dehiscence repair, where conventional suturing repeatedly failed due to extreme tissue fragility. The surgical team ultimately used Quill™ barbed sutures placed perpendicular to the wound to achieve closure without tearing the skin by spreading the tension out over a greater surface. Prior failing attempts with PDS sutures were likened to a “knife through butter”.^26^ Similarly, in a case of vestibuloplasty, sutures were placed far from the edge of the wound to prevent tearing through the mucosa.^27^ Other noted strategies include the use of cylindrical needles to reduce soft tissue trauma.^28^ manual skin harvesting with thin–thickness to prevent delayed epithelialization, Goulian guards to prevent deep cuts caused by excessive mechanical stress produced by auto–driven devices.^29^ generous suturing.^17^ and vascular ligatures over classical hemostasis with multi–layered sutures to accommodate compromised dermal strength.^30^ Other studies reiterated the importance of delicate tissue handling and multilayered suturing, while further emphasizing that the use of skin retractors and dissection should be limited to the necessary extent, with meticulous attention to hemostasis.^31^ Wound edges should be maximally perfused with strict avoidance of unnecessary or excessive compression or tension.^32^ to avoid repeated dehiscence.^26^ and tissue disintegration.^33^

Although direct comparisons remain limited, the available matched cohort studies found no significant rise in complications when patients with EDS underwent elective plastic surgery, suggesting that with meticulous technique outcomes may be equivalent to those of non–EDS controls.^5^^,^^7^^,^^8^ However, other research showed higher rates of hematoma, dehiscence, and delayed healing, particularly with the utilization of standard closure techniques with modifications.^24^ Overall, the literature supports the need for precise, patient–specific preoperative planning in patients with EDS. Tension–reducing measures, vascularity preservation, and avoidance of trauma (e.g., layered closure, flap coverage, and appropriate selection of suture) appear to counteract the inherent risks in this patient group. The diversity and novelty of techniques across cases underscore the critical nature of prior planning and intraoperative adaptability. These strategies represent recurring technical approaches described across case reports rather than evidence–based best practices, as no comparative or controlled data were available to evaluate their effectiveness.

### Sutures and techniques

The most commonly mentioned suture types were Vicryl, Monocryl, PDS, Prolene, and nylon. Stitch techniques were often unspecified, but when noted, included: running subcuticular, horizontal mattress, simple interrupted, and “U” and “O” sutures. The sutures' strength was not routinely reported, and the size varied between 2–0 and 6–0, when named. Closure techniques emphasized layered closure and tension–free approximation. Dressing and wound–support strategies included adhesive–strip or tape support in 8 operative cases (12.3%), specialized or prolonged dressings in 6 (9.2%), and negative–pressure wound therapy in 4 (6.2%) (Table 3).

### Postoperative management

Postoperative wound care was discussed, but to varying degrees of detail. The most commonly mentioned techniques were the use of adhesive support such as steri–strips, micropore tape, and dermabond to stabilize wound edges and reduce tension.^34^^,^^35^ Some protocols delayed the first dressing change to minimize trauma to fragile skin, while others used negative pressure wound therapy on high–risk wounds to promote healing and avoid dehiscence.^36^ Collagen dressings were used in delayed healing or necrosis, said to promote epithelialization.^37^

Activity restrictions were noted and most frequently comprised immobilization with splints or braces, weight–bearing limitation, or avoidance of strenuous activity up to three months postoperatively. Examples included hinged elbow braces, short–arm casts, and surgical bras for stabilization of healing tissues.^18^^,^^38^ These restrictions were instituted to limit mechanical tension on healing tissues and prevent dehiscence or hematoma complications.

Postoperative optimization was mentioned and included interventions such as silicone sheeting for scar management.^35^ nutritional supplementation, and vitamin supplementation (e.g., Vitamin C) as a general guideline.^32^ In some cases, follow–up physical therapy was used to support recovery and monitor joint stability.^18^ Routine follow–up was standard in most cases and occurred over several months to monitor healing and modify care plans based on patient response.

### Postoperative complications

At least one complication was reported in 32 of 77 cases (41.6%; 31 of 65 operative cases, 47.7%). The most frequently reported categories were wound dehiscence (6), delayed or impaired wound healing (6), iatrogenic skin tear or laceration (5), hematoma (5), and bleeding (5), followed by scar abnormality (4), infection (3), and seroma (3); serious systemic events were uncommon (2) (Table 4). Postoperative complications were reported in the majority of studies, but the degree of detail and specificity was inconsistent. In several cases, complications were attributed directly to the tissue fragility characteristic of EDS. In a case of wound dehiscence following aortic surgery, for example, standard suturing techniques routinely failed, and Quill™ barbed sutures implanted perpendicular to the wound were used to achieve closure without tearing the skin.^26^ Another case report cited several hematomas and persistent ecchymosis following cervicofacial rhytidoplasty, with the patient requiring multiple operative visits for drainage and delayed healing.^24^

**Table 4:** Reported postoperative complication categories in the pooled cohort.

Complication category	Cases, n
Wound dehiscence	6
Delayed / impaired wound healing	6
Iatrogenic skin tear / laceration	5
Hematoma	5
Bleeding	5
Scar abnormality	4
Infection	3
Seroma	3
Recurrence	1
Reoperation / revision	2
Serious systemic event	2
Skin necrosis	1
Capsular contracture	1
Other	13

Data are the number of cases reporting each category; categories are not mutually exclusive. "Other" denotes isolated events not otherwise classified.

Despite these challenges, various studies reported uneventful recoveries or no complications, particularly when proper surgical technique and postoperative management were employed. In a case of bilateral reduction mammoplasty for an adolescent with hEDS, the patient experienced no wound issues and was able to resume normal activities with an excellent cosmetic result.^35^ Similarly, in a case of bilateral arthroscopic tendon interposition arthroplasty, the subject had full return to function and resolution of symptoms without any complication.^39^

Two matched cohort studies directly compared complication rates between EDS and non–EDS controls. Fodor et al. found no significant difference in overall (25%vs 35%; P = 0.16) or major (6%vs 12%; P = 0.20) complications, and Najafian et al. reported comparable major–complication rates (2.8%vs 5.4%; P = 0.63) with significantly fewer wound–healing complications among EDS patients (28%vs 47%; P = 0.04), suggesting that with appropriate precautions outcomes may approximate those of non–EDS surgical controls.^8^^,^^57^

When reported, complications appeared more frequently and severe in patients with classical and vascular subtypes associated with tissue and vascular fragility. There existed inconsistent subtype reporting and small sample sizes which limited definitive subtype comparisons. Overall, while an increased risk of wound complications is more probable to arise in patients with EDS, the literature suggests that with proper preoperative planning, meticulous tissue handling, and individually tailored postoperative management, most of these risks can be mitigated and successful outcomes can be obtained.

## Discussion

This narrative review highlights the unique challenges and concerns of managing patients with EDS who undergo plastic and reconstructive surgery. Analysis of 77 cases across 52 studies highlights the importance of subtype–specific risk stratification, meticulous intraoperative technique, and individualized postoperative management to mitigate the inherent risks associated with connective tissue fragility. The patient cohort demonstrated female predominance and a broad age distribution, with hypermobile, classical, and vascular subtypes of EDS being the most commonly represented. Despite the diversity of surgical interventions and specialties presented, reconstructive surgery was the most common indication, reflecting the functional and reparative nature of the tissue complications most frequently associated with EDS.

Among the principal conclusions of this review is the underreporting of preoperative optimization strategies. This gap may reflect limited awareness, time constraints, or publication bias. Few cases reported formal preoperative planning, such as multidisciplinary assessment, hematologic preparation, or physical therapy. This suggests a need for standardized protocols that involve risk stratification and optimization, particularly given the known vascular and healing complications in patients with EDS. Intraoperative tissue handling was a critical determinant of operative success. Literature consistently emphasized tension–free closure, vascularized flap coverage, and avoidance of mechanical stress. Techniques such as modified rhytidectomy, use of barbed sutures, and pre–cord suture placement were used to adjust for tissue friability. Of note, many papers presented novel techniques, including use of trapezius flap in occipitocervical fusion and perpendicular barbed sutures in wound dehiscence repair, underscoring the need for surgical adaptability and innovation in this high–risk population.

An important interpretive caveat concerns the apparent frequency of complications. Within the pooled case–level cohort, at least one complication was reported in 47.7% of operative cases; however, this should not be read as a true complication rate. Case reports and small series are preferentially published when an instructive complication or technical challenge occurs, so the case–level literature is inherently enriched for adverse events. The controlled cohort studies, which compared EDS patients against non–EDS controls, provide more representative estimates: Fodor et al. reported an overall complication rate of 25% (19% minor, 6% major), with wound–dehiscence reoperation actually higher among controls; Najafian et al. found fewer major complications in EDS patients than controls (2.8%vs 5.4%) and fewer wound–healing complications (28%vs 47%).^8^^,^^57^ Norman et al., in a higher–risk craniocervical population, reported infection, dehiscence, and reoperation in 17.6%, 11.8%, and 23.5%, respectively.^22^ Taken together, these comparative data suggest that the complication burden implied by the case–report literature overstates the risk faced by most patients with common subtypes like hEDS/cEDS, and that with appropriate precautions outcomes may approximate those of non–EDS controls.

Surgical risk appears to vary across EDS subtypes. Hypermobile EDS is generally associated with more favorable surgical outcomes, whereas classical EDS carries increased risk of wound complications due to dermal fragility. Vascular EDS represents the highest–risk subtype because of its susceptibility to vascular injury, hemorrhage, and poor tissue integrity, warranting heightened perioperative caution. Although subtype–specific outcome reporting remains inconsistent and robust comparative data are lacking, the reviewed literature supports several recurring perioperative considerations. Preoperatively, identification of EDS subtype and appropriate risk counseling are important, with multidisciplinary evaluation considered for higher–risk patients, particularly those with vascular EDS. Intraoperatively, commonly reported adaptations include tension–free multilayered closure, gentle tissue handling, minimization of tissue undermining and repeated needle passes, preservation of vascularity through atraumatic dissection, and use of flap coverage when necessary. Wider suture bites, slowly absorbable or non–absorbable sutures, and adhesive support may further reduce wound tension. Postoperatively, prolonged wound support, delayed suture removal, activity restriction or immobilization, and close follow–up may help identify early wound–healing complications. These recommendations represent recurring themes from case–level evidence rather than validated best–practice guidelines.

The review is limited in several respects. The sample size was limited to 77 cases from 52 studies, and the majority were single case reports with no control groups or standardized outcome measures. The existing literature consists predominantly of case reports and series. As higher–level comparative data are scarce, particularly among this topic, synthesizing this case–level evidence is essential and represents a feasible approach for characterizing surgical practices in this population. Language bias exists as only English studies were included. Heterogeneity of operation and inconsistent reporting of subtypes of EDS prevented subtype–specific conclusions from being drawn. Pre– and postoperative care routines were sparsely reported, and other significant operative factors such as suture material and method were not consistently documented. Additionally, the retrospective nature of certain studies may introduce potential bias, and publication bias may have favored the reporting of successful or novel cases. These collectively limit the external validity of the findings and underscore the need for superior, prospective studies in this context. Consistent themes across reports represent recurrent observations in low–level evidence, rather than validated clinical recommendations. The predominance of single–patient case reports and small case series precludes any causal inferences regarding the effectiveness of various techniques but rather, the findings of the review identify patterns in how plastic surgeons have adapted to tissue fragility in patients with EDS, highlighting areas where future prospective or comparative studies are needed.

Overall, this review reveals the complexity of and potential for outcome improvements in surgical interventions in patients with EDS. It suggests the need for greater uniformity in reporting preoperative and postoperative procedures, encourages the adoption of EDS–specific operative modifications, and urges the production of evidence–based guidelines to deliver standardized care. Future prospective studies and comparative studies should be directed to further refine best practices and improve outcomes in this high–risk patient population.

## Conclusion

Across studies, commonly described approaches (e.g. tension reduction, vascular preservation, gentle tissue handling, prolonged wound support) reflect shared adaptations within the field to approach the underlying tissue fragility in this demographic. However, the absence of standardized outcome measures, comparative data, and consistent reporting limits the ability to determine the effectiveness of these strategies. Surgical risk varies across subtypes of EDS, with the vascular type carrying greatest operative threats, underscoring a need for subtype–specific risk assessment with perioperative planning.

Favorable outcomes are frequently reported when these techniques are employed, though complication rates remain variable and the risks differ across subtypes of EDS. These findings underscore the important of patient–individualized surgical planning with cautious perioperative management, particularly those in high–risk subtypes, and provide practical considerations that may inform plastic surgical decision making.

Ultimately, this review highlights an existing need within the world of plastic and reconstructive surgery for standardized reporting and prospective studies that better define evidence–based strategies to improve surgical outcomes in this complex patient demographic.

## Declaration of AI and AI–assisted technologies in the writing process statement

During the preparation of this work the author(s) used Microsoft Copilot in order to solely improve the readability of only certain portions of the manuscript, not to generate any unoriginal texts or interpretations of findings, merely modify existing writings as appropriate. After using this tool/service, the author(s) reviewed and edited the content as needed and take(s) full responsibility for the content of the publication.

## Ethical approval

Not required.

## Funding

None.

## Declaration of competing interest

None declared.

## Appendix Supplementary materials

Image, application 1
