# Supplementary material for: Ehlers–Danlos syndrome, an underreported consideration in plastic surgery: A narrative review of cases
Source: JPRAS Open. 2026 Jul 24;51:594–610. doi: 10.1016/j.jpra.2026.07.027 (PMC13485608; doi:10.1016/j.jpra.2026.07.027)
Supplement: Supplementary file 1 [file mmc1.docx]

**Supplementary Table S1.** Per-case data for the published studies summarized in Table 1 (plastic and reconstructive surgical management of Ehlers–Danlos syndrome), ordered chronologically by study date.

| **Author/ Pub. Date** | **Study Type** | **No. of Cases** | **EDS Subtype** | **Age** | **Sex** | **Anatomical Region** | **Indication** | **Reported Treatment Optimizations** | **Complications** | **Key Author Conclusions** |
| --- | --- | --- | --- | --- | --- | --- | --- | --- | --- | --- |
| Ricketson (1957) | Case Report | 1 | NR | 18 | F | Lower Extremity | Reconstructive | Split-thickness skin grafts fixed with interrupted plus a continuous mid-portion mattress suture and pressure dressings; gentle handling with only light traction on tissue forceps, loose sutures, and subcutaneous, subcuticular, and interrupted skin sutures to prevent cut-through; all tension eliminated and the knee splinted in extension; procedures staged | Marked intraoperative skin friability; delayed wound healing; hyperpigmentation of knee scars and within the graft on follow-up | Adequate healing of skin grafts and donor sites does occur in EDS; a patient with EDS may present many and varied surgical problems requiring specialized surgical care for repair |
| Reidy (1963) | Case series | 7 | cEDS | 24 | M | Hand/Upper Extremity | Reconstructive | NR | Delayed donor-site healing | Close wounds early with mattress sutures and adhesive support; deep catgut gives only loose opposition; fine scars are hard to achieve as atrophic collagen holds sutures poorly ("like sewing two bits of porridge"); skin grafts succeed but donor sites heal slowly |
|  | | | cEDS | 14 | NR | Craniofacial; Lower Extremity | Aesthetic | Layered closure, prolonged suture retention | NR |  |
|  |  |  | cEDS | 9 | M | Craniofacial | Aesthetic | No undermining, diathermy hemostasis, subcuticular mattress sutures, prolonged suture retention, adhesive/foam support | Marginal scar heaping/oedema and hyperaemia; scars slightly broader |  |
|  |  |  | cEDS | 8 | F | Lower Extremity | Aesthetic | Excision and suture of the broad, thin knee scars. | NR |  |
|  |  |  | cEDS | NR | F | Craniofacial | — | N/A | N/A |  |
|  |  |  | cEDS | NR | M | Craniofacial | — | N/A | N/A |  |
|  |  |  | cEDS | NR | M | NR | — | N/A | N/A |  |
| Beighton & Bull (1970) | Case report | 1 | NR | 14 | F | Lower Extremity | Aesthetic | Electrocautery hemostasis for edematous/friable subcutaneous tissue; knee immobilized in posterior plaster splint with 14 days bed rest; adhesive strips reapplied after suture removal to support the scar | NR | Correct timing of surgery is critical, early adolescence being the optimum period; recognizing the EDS subtype indicative of operative risk and outcome |
| Guerrerosantos & Dicksheet (1985) | Case report | 1 | NR | 62 | F | Craniofacial | Aesthetic | Local anesthesia and IV sedation; SMAS/platysma plication abandoned intraoperatively (ligatures cut through friable tissue); prolonged bulky compression dressing (~3 wk) | Recurrent large cervicofacial hematomas; retroauricular wound dehiscence with wide, depressed scars; residual submental flaccidity, periorbital edema | EDS poses a formidable threat to facelift surgery; exclude EDS preoperatively in prematurely-wrinkled patients seeking a facelift. Rhytidoplasty is best discouraged in EDS |
| Moore et al. (1985) | Case series | 2 | cEDS | 15 | F | Hand/Upper Extremity | Reconstructive | Transverse incision in skin crease; cup-in-cone fusion at ~40° palmar abduction; thumb spica immobilization 2 mo | NR | In EDS, predictable bony procedures are preferable to soft-tissue reconstruction, which tends to stretch out |
|  |  |  | cEDS | 15 | M | Hand/Upper Extremity | — | CMC joint splint for symptom relief | N/A | Symptomatic CMC subluxation may be commoner in EDS than appreciated; milder instability can be managed conservatively |
| Borris et al. (1997) | Case report | 1 | vEDS | 44 | M | Craniofacial | Reconstructive | Wide suture placement away from edges, careful hemostasis, custom acrylic stent fixation, prolonged wound care | Suture granulomas; delayed donor-site epithelialization | With meticulous technique and patience, split-thickness skin grafting can succeed in EDS despite prolonged healing |
| Erçöçen et al. (1997) | Case report | 1 | cEDS | 20 | M | Hand/Upper Extremity | Reconstructive | No surgical intervention undertaken; a Silver Ring splint offered for the affected fingers, but the patient declined it | NR | The potential hazards of surgery make surgery inappropriate for hand deformities in EDS, favoring conservative management EDS |
| Oliver et al. (2000) | Prospective study | 6 | hEDS 3, cEDS 2, vEDS 1 | 25-63 | M (4)  F (2) | Multisite | Other | NR | NR | Local anaesthetic resistance in EDS is not due to rapid tissue dispersal; increasing dose is unlikely to overcome resistance |
| Badia et al. (2005) | Case report | 1 | NR | 56 | M | Hand/Upper Extremity | Reconstructive | Arthroscopic approach, tendon interposition using APL slip, thermal capsular shrinkage, trapezial bur abrasion for graft adhesion, thumb spica immobilization, staged bilateral procedures | NR | A joint-preserving arthroscopic interposition arthroplasty is a viable alternative to the traditionally recommended arthrodesis for EDS basal-joint arthritis. It preserves thumb motion, relieved pain and restored strength, and does not preclude later revision if laxity recurs. |
| Mueller et al. (2005) | Case report | 1 | cEDS | 61 | F | Craniofacial | Aesthetic | Staged full-face treatment at 9-month interval, pulsed CO₂ then CO₂/erbium:YAG combination laser, postop acyclovir + sultamicillin prophylaxis | NR | Laser resurfacing gave better, durable results than the prior facelifts in severe EDS and is less-invasive when surgery is too risky; the CO₂/erbium:YAG combination laser matched the CO₂ laser's effect with markedly faster healing and less erythema |
| Rossi & Kappel (2006) | Case report | 1 | NR | 40 | F | Craniofacial | Reconstructive | Vascularized temporalis osteofascial flap as a functional disk replacement, bone-to-bone interosseous wire/screw fixation avoiding soft-tissue reliance, fossa-edge bone flap, condyle recontouring, 6-week occlusal splint + intermaxillary fixation. | Disk displacement at 2.5 years; tenderness over wire | Bone-to-bone fixation superior to soft-tissue reconstruction in EDS due to unpredictable wound healing. |
| Tan et al. (2009) | Case report | 1 | aEDS | 17 | F | Craniofacial | Reconstructive | Surgery deferred due to EDS anesthetic/surgical risk; conservative referral to occupational therapy for leg braces and padding of pressure-point molluscoid pseudotumors | NR | EDS may be associated with cleft lip/palate via a possible but not-yet-elucidated connective-tissue failure-of-fusion mechanism; more case reports needed to establish the link. |
| Whitaker et al. (2009) | Case report | 1 | aEDS | 5 | M | Trunk/Abdomen | Reconstructive | Combined plasticorthopedic approach; two-layer closure (4/0 Vicryl and Monocryl); Steri-strips and Mepore; prolonged Micropore tape (3 months); avoidance of adhesive electrodes | Laceration from adhesive electrode; ecchymoses with minimal pressure; erythema from dressing; postoperative anaemia requiring 340 mL transfusion; slight lower-scar stretching | Plastic surgery involvement recommended for all EDS Type VII cases undergoing surgery. Meticulous wound management with prolonged supportive measures essential |
| Cheung et al. (2010) | Case report | 1 | cEDS | 17 | F | Hand/Upper Extremity | Reconstructive | Targeted excision of frayed FDP/FDS tendon fibrils; A1 pulley preserved; intraoperative tendon-excursion testing to confirm smooth gliding; elimination of precipitating repetitive microtrauma | NR | Multiple triggering can occur in EDS from tendon degeneration/fibril fraying (repetitive microtrauma) rather than pulley stenosis; treatment need not divide the A1 pulley |
| Kumar et al. (2010) | Case report | 1 | vEDS | 22 | M | Lower Extremity | Reconstructive | Limited access dressing (intermittent negative-pressure) applied over both recipient and donor sites; 3-week wound-bed preparation before grafting | Donor area cut deeper than intended at the usual Humby-knife setting due to skin laxity | Intermittent negative-pressure dressing produced prompt healing, superior to the conventional closed dressing that had previously delayed healing in this patient |
| de Weerd et al. (2012) | Case report | 1 | vEDS | 56 | F | Trunk/Abdomen | Reconstructive | Open approach for gentle handling; tension-free retromuscular mesh; peritoneum interposed between mesh and bowel; temporary VAC stabilization, sponge wrapped to reduce bleeding at changes; staged second mesh after fibrosis; hernia belt | small stable residual bulge, no recurrence at 2-yr follow-up | Complex abdominal wall hernia in vascular EDS can be managed with staged, tension-free mesh reconstruction; temporary negative-pressure stabilization holds the wall until the mesh/tissue incorporates and may suit other complex reconstructions |
| de Weerd et al. (2012) | Case report | 1 | hEDS | 47 | F | Trunk/Abdomen | Reconstructive | Vascularized autologous tissue instead of mesh, muscle-sparing harvest, flap fixation to prevent descent, postoperative hernia belt | NR | A vascularized ms-TRAM "dam" can treat enterocele in EDS without synthetic mesh and with little donor-site morbidity, avoiding mesh hazards related to tissue friability |
| Kelley & Heller (2012) | Case report | 1 | vEDS | 31 | F | Trunk/Abdomen | Reconstructive | Delayed closure after infection control; biologic mesh onlay bridging fascial defect, secured with atraumatic care; wide bilateral skin undermining; Quill barbed sutures placed perpendicular to wound to diffuse tension away from friable edges | Intra-abdominal hematoma with fascial/skin dehiscence; polymicrobial infection; recurrent skin tearing with edge lacerations | Distributing tension away from friable wound edges via perpendicular barbed sutures achieves closure where conventional edge-tension techniques fail; applicable to any patient at high risk of dehiscence |
| Ryan et al. (2012) | Case report | 1 | hEDS | 17 | F | Trunk/Abdomen | Reconstructive | Conservative wound management: topical clobetasol steroids, PHMB bio-cellulose dressing 3×/week, empirical vitamin C 5000 U/day, multidisciplinary care | Recurrent wound dehiscence over ~3 years | Consider EDS in unexplained/prolonged wound dehiscence as early recognition avoids repeated invasive procedures and harmful misdiagnosis and enables successful conservative healing; refer such patients for rheumatology review |
| Bouhassira et al. (2014) | Case report | 1 | hEDS | 28 | F | Breast;  Trunk/Abdomen | Aesthetic | Multistage approach; tension-free/multilevel closure; moderate skin resection to minimize tension; vascular ligatures over classic hemostasis; delayed suture removal; compatible PRBC on hand | Wound dehiscences, seroma, postoperative hematoma requiring transfusion and revision, umbilical ascent | Body-contouring in EDS carries high risk of dehiscence and bleeding with only modest aesthetic gains; reserve for patients with major daily impairment who accept the risks and technical difficulty |
| Busch et al. (2014) | Case report | 1 | kEDS | 32 | M | Lower Extremity | Reconstructive | Open wound healing with VAC therapy; delayed split-thickness mesh skin graft for impaired groin wound | Impaired groin healing; failed skin closure with serous effusion, necessitating open wound management | In EDS, buttressed/primary skin closure often fails due to heavy serous oozing; open wound management with vacuum therapy followed by meshed split-thickness skin grafting achieves reliable healing at donor and recipient sites |
| Krijgh et al. (2014) | Case series | 6 | hEDS | 20 | F | Hand/Upper Extremity | Reconstructive | Autologous ECRB tendon stabilization; low tendon tension to avoid dislocating the lax lunate; 12-week immobilization | NR | ECRB tenodesis may offer a surgical solution to recurrent midcarpal instability in EDS-HT, reducing pain, luxations and orthotic reliance; autologous tendon appears feasible despite abnormal collagen metabolism, though longer follow-up is needed for durability |
|  | | | hEDS | 22 | F | Hand/Upper Extremity | Reconstructive | Combined tendon stabilization; prolonged immobilization | NR |  |
|  |  |  | hEDS | 38 | F | Hand/Upper Extremity | Reconstructive | Autologous tendon stabilization | Lunate/capitate dislocation |  |
|  |  |  | hEDS | 31 | F | Hand/Upper Extremity | Reconstructive | Autologous tendon stabilization; immobilization | NR |  |
|  |  |  | hEDS | 32 | F | Hand/Upper Extremity | Reconstructive | Autologous tendon stabilization; immobilization | NR |  |
|  |  |  | hEDS | 28 | F | Hand/Upper Extremity | Reconstructive | Autologous tendon stabilization | Prolonged postoperative edema |  |
| Creasy et al. (2016) | Case report | 1 | cEDS | 44 | F | Breast | Reconstructive | Gentle vessel handling at anastomoses avoiding multiple needle passes/intimal damage; meticulous hemostasis; tension-free multilayered closure; careful intraoperative positioning, pressure-area protection | Small periumbilical hernia | Classical EDS is not a contraindication to microsurgical breast reconstruction; applicability to other subtypes is unclear; EDS patients may benefit from routine mesh reinforcement of the rectus sheath |
| Harrison et al. (2016) | Systematic review | — | Multiple Types | NR | NR | Trunk/Abdomen | Reconstructive | Components separation technique; polypropylene mesh overlay; careful tissue handling;  biologic mesh theoretic benefit but possibly altered response in collagen-deficient patients; experimental adjuncts | High hernia recurrence risk; wound dehiscence; poor scarring; intra/postoperative bleeding | High-level data linking collagenopathies to hernia are lacking, but pathologic trends support a collagen production/processing defect contributing to direct, indirect and ventral hernias; surgeons should mechanically optimize repair and counsel patients on the high recurrence risk |
| Lindsay et al. (2016) | Case Report | 1 | hEDS | 13 | F | Craniofacial | Reconstructive | Perioperative tranexamic acid, recombinant factor VIIa, meticulous tissue handling, generous suturing | Mild postoperative oozing | Bleeding risk is common in EDS but prophylaxis is unstandardized; a preoperative bleeding workup with targeted hemostatic prophylaxis can prevent excessive perioperative bleeding. |
| Rollett et al. (2016) | Case report | 1 | cEDS | 55 | F | Craniofacial | Aesthetic | Modified MACS facelift with no skin undermining; limited tissue excision; deep lifting sutures anchored to fixed points; meticulous hemostasis; two-layer closure with absorbable sutures; steri-strips and gentle compression bandage | Severe bruising at IV cannulation site | Facelift is feasible in selected EDS patients when invasiveness is minimized and tension is transferred to deep support sutures rather than the skin, yielding a natural, stable result without the complications historically expected |
| Ericson & Wolman (2017) | Expert consensus | — | Multiple  Types | NR | NR | Multisite | Reconstructive | Exhaust non-operative care first; smaller incisions; tendon/soft-tissue stabilization preferred; wrist & thumb stabilization favored; nerve decompression; Botox for TOS; ropivacaine/carbocaine over lidocaine/bupivacaine | Higher recurrence of joint stabilization; wider scars; less predictable healing/bleeding | Non-operative treatment is preferable, but for carefully selected, correctly diagnosed patients, specific joint-stabilization and nerve-decompression procedures can give reliable symptomatic relief when conservative measures fail |
| Joseph et al. (2017) | Systematic review | — | Multiple Types | NR | NR | Craniofacial | Aesthetic; Reconstructive | Gentle tissue handling; meticulous hemostasis, avoid vessel loops/clamps and skin staples/hooks; layered tension-minimizing closure; Steri-strip/tape support; prolonged suture retention; consider non-invasive alternatives; preop cross and type and coagulopathy workup | Wound dehiscence, infection, hematoma, poor scarring, excessive bleeding | Vascular EDS is a contraindication to elective surgery; classic and hypermobile EDS can typically undergo facial plastic surgery without major complications when identified preoperatively and managed with individualized, extreme-care technique |
| Hosomi et al. (2018) | Case report | 1 | NR | 17 | F | Lower Extremity | Reconstructive | Ultra-thin manual harvest; small-slice “net” pattern preserving skin islands; epinephrine/saline-soaked gauze for hemostasis; non-adherent silicone mesh primary dressing; switch to hydrocolloid at day 5–7; dressing change every 2–3 days; staged repeat harvest from same donor site | NR | Net-pattern small-slice thin-thickness harvesting leaves abundant keratinocytes and net-patterned dermis, enabling non-delayed, stable epithelialization and safe repeat grafting from the same donor site despite EDS skin fragility |
| Baik et al. (2019) | Case report | 1 | NR | 41 | M | Lower Extremity | Reconstructive | One-stage elliptical excision chosen to exploit hyperextensibility; suprafascial-level excision; subcutaneous undermining of margins; layered closure; prolonged suture retention/delayed removal; collagen-alginate wound dressing changed every 3 days | Mild wound discharge; partial wound dehiscence; partial skin necrosis from added bolster stitches; overall delayed healing | Recognizing EDS lets the surgeon anticipate poor healing and adjust the reconstructive plan to reduce morbidity; one-stage closure is feasible given hyperextensibility when the wound is not too wide; remove skin sutures ≥5 days later than in non-EDS; a mesh overlay graft for musculofascial support is preferable to bolster sutures; collagen dressing may accelerate healing |
| Hevesi et al. (2019) | Retrospective cohort | 9 | hEDS | 61 | F | Hand/Upper Extremity | Reconstructive | Concurrent MCP fusion + suspensionplasty | NR | Operative stabilization can yield durable pain relief in EDS-related CMC arthritis  Minimally invasive techniques can improve symptoms in selected EDS patients  Soft-tissue reconstruction may be required after implant failure in EDS  Autologous ligament reconstruction provides acceptable outcomes in EDS |
|  | | | hEDS | 41 | M | Hand/Upper Extremity | Reconstructive | Joint-preserving arthroscopy with capsular shrinkage | NR |  |
|  |  |  | hEDS | 64 | F | Hand/Upper Extremity | Reconstructive | Soft-tissue allograft interposition after implant failure | Revision surgery required |  |
|  |  |  | hEDS | 52 | M | Hand/Upper Extremity | Reconstructive | Autologous tendon interposition | NR |  |
|  |  |  | hEDS | x̄ = 49 | NR | Hand/Upper Extremity | — | Rest, NSAIDs/topical diclofenac, splints, steroid injections | N/A |  |
| Schneider et al. (2019) | Case report | 1 | NR | 22 | F | Hand/Upper Extremity | Reconstructive | Allograft ligament reconstruction, temporary hinged internal fixation, shared lateral isometric point for axis pin and LCL origin, unicortical axis-pin drilling, early protected motion | NR | Collateral ligament reconstruction with allograft plus a temporary hinged internal fixator is a viable option to achieve stability, restore function, and preserve quality of life in EDS-related recurrent elbow instability |
| Shaharan et al. (2019) | Case report | 1 | NR | 5 | NR | Lower Extremity | Reconstructive | Longitudinal Steri-Strips applied parallel to wound, tape reinforcement at wound edges, horizontal mattress sutures over strips, delayed suture removal at 2 weeks | NR | Suture/adhesive-strip combination is not limited to elderly thin skin and has wider applications for fragile-skinned patients such as those with EDS |
| Xu et al. (2019) | Case report | 1 | cEDS | 30 | F | Craniofacial | Aesthetic | Tension minimization, precise wound edge apposition | NR | cEDS, scar revision that minimizes wound tension and achieves precise dermal apposition can yield a durable fine-line result |
| Lu et al. (2020) | Retrospective cohort | 19^b^ | NR | NR | NR | Craniofacial | Reconstructive | Well-vascularized myofascial (paraspinous muscle) flap closure after craniocervical decompression/fusion | Infection; wound breakdown; reoperation | Myofascial flap closure is safe in EDS and may reduce reoperation after craniocervical surgery though in the EDS subgroup the reduction was only a non-significant trend |
| Scheufler et al. (2020) | Case report | 2 | cEDS | 43 | F | Trunk/Abdomen | Reconstructive | Extended two-layer rectus-aponeurosis plication, onlay VIPRO-mesh (xiphoid–symphysis); postoperative abdominal belt ×3 mo, delayed muscle-building exercise | NR | Mesh-reinforced abdominoplasty with rectus plication offers safe, durable abdominal-wall reconstruction in cEDS with lasting functional benefit. |
|  |  |  | cEDS | 50 | F | Trunk/Abdomen | Reconstructive | Open repair of superior and inferior lumbar hernias with VIPRO-mesh insertion; muscles closed over mesh in a double layer | NR |  |
| Deshpande et al. (2021) | Case report | 1 | hEDS | 62 | F | Breast | Reconstructive | Goldilocks mastectomy (preserved inferior dermoglandular pedicle); de-epithelialization of dermal flaps; proton therapy; preoperative EDS-specialist cardiac/anesthesia clearance and PM&R lymphedema screening | Grade II dermatitis one month after radiation | Goldilocks mastectomy is an excellent reconstructive option in EDS patients predisposed to poor wound healing, and adjuvant radiation can be tolerated when carefully designed; management must be tailored to the individual's hEDS variant and comorbidities |
| Najafian et al. (2022) | Retrospective cohort | 36^a^ | hEDS 26, cEDS 1, Unspecified 9 | x̃ =  27 | M (12)  F (24) | Multisite | Reconstructive | Standard surgical techniques; careful postoperative monitoring; no EDS-specific protocol deviation reported | Major complications: 2.8% (EDS) vs 5.4% (controls); wound-healing issues: 28% (EDS) vs 47% (controls) | An EDS diagnosis alone should not be a barrier to gender-affirming surgery; complication and reoperation rates were comparable to (or better than) non-EDS controls |
| Norman et al. (2022) | Retrospective cohort | 34^b^ | NR | x̄ =  23.0 | M (11)  F (23) | Craniofacial | Reconstructive | Paraspinous muscle release and transposition over hardware/bone graft, imbricated to obliterate dead space; trapezius myofascial advancement to cover occipital plate; watertight subfascial seal over dura; drains | infection 17.6%, dehiscence 11.8%, reoperation 23.5%, 1 pseudomeningocele | Myofascial flap closure is safe in high-risk EDS Chiari patients and reduces complications after complex craniocervical surgery to the level of simple surgery, while preventing CSF cutaneous fistula and pseudomeningocele progression |
| Padmanabhan et al. (2022) | Case report | 1 | cEDS | 59 | F | Breast | Reconstructive | Complete capsulectomy, exchange to smooth implant | Baker IV capsular contracture and pain | Local mechanical stress from a textured implant can override the EDS collagen defect, producing an atypically organized, inflamed internal capsule mechanistically linking textured surfaces to prolonged inflammation and BIA-ALCL risk |
| Hasegawa et al. (2023) | Case report | 1 | vEDS | 46 | M | Lower Extremity | Reconstructive | Preop CTA + Doppler perforator mapping; pedicled posterior tibial a. perforator flap; maximal soft-tissue preservation around the perforator to avoid mechanical stress on fragile vessels; staged reconstruction with artificial dermis then full-thickness skin graft | NR | Pedicled perforator flap reconstruction can be safely performed in vascular EDS with meticulous planning and minimized invasiveness |
| Ishimoto et al. (2023) | Case report | 1 | hEDS | 18 | F | Breast | Reconstructive | Wise-pattern inferior-pedicle reduction; two-layer dermal closure, no drains, prolonged Steri-Strips, silicone scar therapy; multidisciplinary preop | NR | First reported adolescent breast reduction in EDS; hEDS is not a contraindication and these patients may benefit more given worse macromastia symptoms |
| Lellouch et al. (2023) | Case report | 1 | vEDS | 27 | M | Trunk/Abdomen | Reconstructive | Two-stage free latissimus dorsi flap transferred to groin, folded on itself and monitored 8 days to confirm viability before visceral repair and abdominal closure; muscle around pedicle left to heal by secondary intention to avoid tension; LMWH thromboprophylaxis | Enterocutaneous fistula recurrence | Staging the free flap lets viability be confirmed before visceral closure, so any flap failure won't expose viscera; thus, enabling safe full-thickness abdominal wall restoration in high-risk vascular EDS |
| Shomorony et al. (2023) | Case report | 1 | cEDS;  hEDS | 25 | F | Craniofacial | Reconstructive | Endonasal approach, quilting sutures with polyglactin, septal splints, careful tissue handling | Postoperative TMJ dislocations, nausea/vomiting | Septorhinoplasty can be performed successfully in EDS with modified technique and multidisciplinary perioperative management |
| Wanderley et al. (2023) | Case report | 1 | dEDS | 3 | M | Craniofacial | Reconstructive | Vascularized pericranium flap for dural closure; split autologous cranial bone graft fixed with steel wire for cranioplasty; cylindrical needles for atraumatic soft-tissue closure; multidisciplinary approach with plastic surgery | NR | First reported association of growing skull fracture with EDS; intraoperative dural fragility prevented direct suture repair. Early MRI should be considered in pediatric EDS patients with skull fractures to detect dural tears |
| Angwin et al. (2024) | Expert consensus | — | Multiple Types | NR | NR | Multisite | Other | Injury prevention; tension-free 2-layer closure with generous deep sutures left 2× longer; least-reactive monofilament sutures, avoid silk, catgut, and mattress sutures; cyanoacrylate ± adhesive skin tapes for small wounds; nonadherent dressings removed opposite the injury direction; TXA/DDAVP for bleeding/haematoma | NR | Injury prevention and tension-minimizing, low-adherence wound care are the core principles across all EDS types; evidence base is poor and a formal Delphi consensus is needed |
| Morris et al. (2024) | Case series | 4 | hEDS | 22 | F | Breast | Aesthetic | meticulous hemostasis, tension-free closure, careful positioning/pressure | Suture spitting | hEDS diagnosis is not a contraindication to breast surgery. Acceptable outcomes achievable with proper technique. Even large resection volumes well-tolerated. |
|  | | | hEDS | 42 | F | Breast | Reconstructive | NR | NR |  |
|  |  |  | hEDS | 40 | F | Breast | Reconstructive | NR | NR |  |
|  |  |  | hEDS | 18 | F | Breast | Reconstructive | NR | NR |  |
| Nehila et al. (2024) | Case report | 1 | cEDS; vEDS | 41 | F | Breast | Reconstructive | Two-stage reconstruction, meticulous intraoperative hemostasis, conservative implant-based approach | Increased intraoperative bleeding; small seroma | First reported breast reconstruction in combined cEDS/vEDS; Conservative 2-stage tissue-expander approach yielded an unremarkable surgical and postoperative course despite the high-risk subtype combination |
| Angwin et al. (2025) | Case series | 5 | dEDS | 42 | F | Multisite | Reconstructive | NR | Cardiac arrest | First reported adult dEDS cohort; extreme skin fragility persists lifelong, driving high iatrogenic-injury risk. Management needs early plastic-surgery input, low-friction manual handling to prevent tears, low threshold for fracture/bone-density investigation, and MDT care |
|  | | | dEDS | 25 | M | Hand/Upper Extremity | Reconstructive | Closure with adhesive strips & cyanoacrylate glue gave best healing outcomes | Iatrogenic skin tear |  |
|  |  |  | dEDS | 22 | F | Multisite | Reconstructive | Absorbable sutures noted to dissolve poorly; skin handling caution | Iatrogenic skin tears, infection and cellulitis |  |
|  |  |  | dEDS | 25 | M | Multisite | Reconstructive | Adhesive tapes and/or cyanoacrylate glue used for small injuries | Iatrogenic skin lacerations and hematomas |  |
|  |  |  | dEDS | 26 | M | Hand/Upper Extremity | Reconstructive | NR | Unsuccessful childhood camptodactyly surgery |  |
| Fodor et al. (2025) | Retrospective Cohort | 100^a^ | hEDS 41, cEDS 6, vEDS 2, clEDS 1, kEDS 1, mEDS 1; unspecified 48 | x̄ =  43.3 | M (14)  F (86) | Multisite | Aesthetic;  Reconstructive | Individualized preoperative evaluation, subtype determination, and accounting for comorbidities | Overall complications 25%; minor 19%, major 6%; wound-dehiscence reoperation actually higher in controls | Elective plastic surgery can be performed safely in EDS, especially hEDS with complication rates comparable to matched controls; EDS should not be a blanket relative contraindication, and disqualification based on an EDS diagnosis alone should be the exception |
| Rostami et al. (2025) | Case report | 1 | cEDS | 53 | M | Lower Extremity | Reconstructive | Integra wound-bed priming (NPWT + external fixation); thin STSG harvest for fragile cEDS dermis; RECELL spray over 3:1 mesh to spare donor skin; adjunctive testosterone, HGH, hyperbaric O₂ for collagen synthesis/angiogenesis | NR | STSG plus offers an effective modality for closing large complex wounds in cEDS with minimal donor morbidity |
| Ziółkowska et al. (2025) | Case report | 1 | NR | 34 | F | Multisite | Reconstructive | Collagen-based products chosen for the collagen defect; in-vitro cultured autologous keratinocytes/fibroblasts in PRP; micrografting; discontinuation of psychotropics improved healing; LASCA microperfusion monitoring | Septic shock with respiratory failure; prolonged nonhealing wound | Burn wounds in EDS need prolonged, multi-stage treatment with innovative preparations; healing progress can be tracked with LASCA |

ᵃ Retrospective cohort/comparative studies (Najafian 2022, n = 36; Fodor 2025, n = 100) reporting EDS outcomes against non-EDS controls; summarized, not included in the individual-case count.

ᵇ Lu (2020; 19 EDS patients of 24) and Norman (2022; 34 EDS patients of 62) report overlapping cohorts from one institution; summarized as study-level entries and not included in the individual-case count.
